# A case-control study on oral health knowledge and dental behavior among individuals with developmental delays in Jordan: caregiver perspective

**DOI:** 10.3389/fdmed.2024.1426568

**Published:** 2024-11-13

**Authors:** Sabha Mahmoud Alshatrat, Wael Mousa Al-Omari, Abedelmalek Kalefh Tabnjh, Isra Abdulkarim Al-Bakri, Siddharthan Selvaraj

**Affiliations:** ^1^ Department of Applied Dental Sciences, College of Applied Medical Sciences, Jordan University of Science and Technology, Irbid, Jordan; ^2^ Department of Prosthodontics, Faculty of Dentistry, Jordan University of Science and Technology, Irbid, Jordan; ^3^ Department of Cariology, Institute of Odontology, The Sahlgrenska Academy, University of Gothenburg, Gothenburg, Sweden; ^4^ Department of Community Oral Health and Clinical Prevention, Faculty of Dentistry, University of Malaya, Kuala Lumpur, Malaysia; ^5^ Department of Dental Research Cell, Dr. D. Y. Patil Dental College & Hospital, Dr. D. Y. Patil Vidyapeeth, Pune, India; ^6^ Department of Public Health Dentistry, Saveetha Dental College and Hospitals, Saveetha Institute of Medical and Technical Sciences, Saveetha University, Chennai, India

**Keywords:** oral care, dental behavior, oral health knowledge, intellectual and developmental disabilities, special need

## Abstract

**Aim:**

the aim of this study is to assess oral health knowledge and dental behavior among individuals with developmental delays intellectual and developmental disabilities (IDD) in comparison with individuals without (IDD) in Jordan.

**Methods:**

A case–control study was conducted among 317 caregivers of individuals with IDD (*n* = 168) and control (*n* = 149) groups, which involved the completion of the questionnaire. A closed-ended, validated self-designed questionnaire was distributed. The questionnaire included questions addressing participants’ oral health knowledge and behavior. Convenience sampling was used to select the caregivers from centers associated with IDD by collecting data from nearby locations. Using SPSS® software Version 22 with a 0.05 level of significance data were analyzed. A Chi-square test and contingency-table analysis were performed on the data.

**Results:**

Caregivers for individuals with IDD in Jordan were less knowledgeable about different oral health aspects than control group (*P* value < 0.05). Fewer individuals in the IDD caregiver group brushed their teeth once or twice daily (83.2%), compared to the control group (93.3%). Only 22.6% of the IDD participants could brush their teeth without assistance. On the other hand, the IDD group consumed significantly less sugary foods and soda than the control group (*P* value < 0.05).

**Conclusion:**

The lack of knowledge among IDD caregivers in Jordan is critical which can result in poor oral health outcomes for this vulnerable population. Increasing the knowledge level for the IDD caregivers on the specific needs for IDD individuals is highly important to enhance their understanding of oral health and improve their quality of life. Caregivers with IDD should receive training programs given by oral health professionals about the need for regular dental check-up and effective oral hygiene care reduce the risk of having dental problems and oral disease.

## 1 Introduction

Oral health has a large impact on general health and oral health-related quality of life. Most oral diseases are preventable or reduced by good oral hygiene techniques, healthy diet, and effective treatment options (1–3). Good oral health may reduce the risk of oral complications for healthy and special need individuals, such as intellectual and developmental disabilities.

Intellectual and developmental disabilities refer to a delay that affects more than one developmental area, such as social/personal, gross/fine motor, speech/language, cognitive, or daily living tasks (4). There are three categories of developmental delay: mild (functional age is less than 33% of chronological age), moderate (34%–66% of chronological age), and severe (functional age is less than 66% of chronological age) (5).

Health disparities between persons with intellectual and developmental disabilities (IDD) and those without IDD, according to recent research (6–8). People with IDD have worse oral health than those without, according to studies, which raises serious concerns about their oral health (9, 10). Additionally, a comprehensive analysis by Anders and Davis (9) has explicitly demonstrated that, in contrast to those without IDD, those with IDD had a greater frequency of periodontal disease and neglected dental caries. Recent studies have shown that poor dental health can have serious consequences for overall health, including strong correlations with aspiration pneumonia and major chronic illnesses such diabetes, stroke respiratory disease and cardiovascular disease (11–17).

A person's psychological and social wellbeing are significantly impacted by their oral health. For instance, poor dental health can result in toothaches, the anxiety that goes along with them, trouble carrying out daily tasks, poorer social relations, and decreased nutrient intake (18–24). Cognitive impairments are the most common impairment among young people (25). Autism, intellectual disability, anoxic brain damage, stroke, and post-traumatic injury that causes learning problems are a few examples of cognitive disorders (24) One in six children in the US (from 2009 to 2017) between the ages of 3 and 17 were found to have developmental problems, a rise from prior years (24, 26–28).

Disorders that have a detrimental effect on a person's physical, intellectual, and emotional development are known as intellectual and developmental disabilities (IDD). All racial, ethnic, and socioeconomic groups are susceptible to these disorders occurring *in utero*. They can also develop after birth because of trauma, illness, or other environmental factors (29).

Individuals with IDD usually have complicated oral health needs owing to congenital and developmental defects that may be further affected by behavior patterns and communication challenges (9). Patients with IDD require more time, patience, understanding of dental health, training in home care, and general caregivers’ abilities than patients without ID (30).

In addition to the absence of fundamental information about oral health and poor training, frequently describe a lack of time and recalcitrant patients as challenges to delivering oral hygiene treatment to people with IDD (31). The purpose of this study was to measure the oral health knowledge and dental behavior among individuals with developmental delays in Jordan. Regular preventative dental treatment is crucial to preserving excellent oral health since people with disabilities are more susceptible to oral illnesses owing to underlying congenital defects and an inability to get the necessary care to preserve oral health which can be achieved by assessing the level of knowledge and behavior and instilling when its lacking. Therefore, the purpose of this study is to evaluate dental knowledge and behavior among people with developmental disabilities.

## 2 Materials and methods

This study was conducted in accordance with the World Medical Association Declaration of Helsinki (32). To answer the study objectives, a self-designed questionnaire was used in this study. The questionnaire was formulated in the official Jordanian language; the Arabic language. Back translation was conducted to ensure accuracy and consistency of the questionnaire. The Expert panel of two professional colleagues established the content validity of the questionnaire, and the Average Congruency Percentage (ACP) was 92% indicating acceptable application for the questionnaire in this study. The reliability of this study (test-retest) was achieved by administering the questionnaire twice to the same participants (*n* = 10). Cronbach's alpha was used to test the internal reliability of this questionnaire. The coefficient of Cronbach's alpha was 0.75 indicating the items have acceptable internal consistency. A pilot study was made by giving the questionnaire to ten volunteers of caregivers of individuals with IDD to answer the questionnaire on behalf of individuals with IDD and provide feedback on the questionnaire clarity, content, and format, during meeting at the IDD'S centers. The participants of the pilot study were not included in the study. The caregiver comments were taken into consideration for the final study. The Sample Size Calculator software was used in this study and the sample size calculations provided with a confidence level of 95%, a margin of error of 5%.

Three sections were included in the questionnaire: First section consisted of items covered demographic information (5 items), Second section included items covered information about participant's oral-health knowledge (12 items), and the third section included items covered participant's dental behavior (7 items). The degree of disability severity for the participants was provided by the principal of the center which obtained from the participant's record.

A list of the different areas in Jordan was obtained from the Ministry of Social Development contains list of centers associated with IDD. The centers who agreed to participate in the study were included. Convenience sampling was used for this study by collecting data from nearby locations. The researcher visited the centers who are willing to participate and asked to circulate the questionnaire to their members to answer the questions on respondent's behalf. A paper format of the questionnaire was available in these centers with a cover letter that explaining the purpose of the study. A full confidentiality of the obtained data was ensured and the participation of the study was voluntary. Regarding the control group, it was composed of individuals without developmental delay from the same geographic location of the centers who agreed to participate in the study by collecting data from nearby locations such as, schools, mall, and parks. After one to three weeks, the questionnaire was collected. A reminder note was sent for those who had not responded to encourage them to reply. the deadline was set for receiving the questionnaires after six weeks from the initial sending. The number of the questionnaires were sent to all individuals with developmental delay was 200, out of which 168 questionnaires were filled and sent back. However, 149 filled questionnaires from the control group were sent back out of 200 that were sent.

All the data were reported in group form. The software package, SPSS Version 23, was used to analyze the data with a 0.05 level of significance. A chi-square test and contingency table analysis were performed on the data.

## 3 Results

The final sample sizes for IDD and control groups were (*n* = 168) and (*n* = 149), respectively. Data showed that 11.3% of individuals with IDD had a mild disability, 33.9% had a moderate disability, and 54.8% had a severe disability. The average age of the total sample was 37 years. Table 1 provides detailed information regarding the socio-demographic characteristics of participants with IDD and control group.

**Table 1:** Socio-demographic characteristics of participants with developmental delay and control group.

Characteristics	Developmental delay *n* (%)	Control *n* (%)
Gender		
Male	103 (61.3%)	100 (67.1%)
Female	65 (38.7%)	49 (32.9%)
Age		
<18	32 (19%)	97 (65%)
18–40	95 (56.5%)	37 (25%)
>40	41 (24.5%)	15 (10%)
Education		
Elementary	152 (91%)	132 (88.6%)
Middle school	9 (5.4%)	17 (11.4%)
High school	4 (2.4%)	0 (0%)
Collage and more	2 (1.2%)	0 (0%)
Family Income (JD)		
<250	143 (85.1%)	2 (1.3%)
250–500	4 (2.4%)	70 (47.0%)
500–1,000	1 (9.5%)	64 (43.0%)
>1,000	20 (11.9%)	13 (8.7%)
Insurance		
Yes	138 (82.6%)	100 (67.1%)
No	29 (17.4%)	49 (32.9%)

Concerning the responses of the participants to oral-health knowledge, caregivers for individuals with IDD scored significantly lower than the control group on the majority of items (*P* value < 0.05). However, there was no discernible difference in the responses to the questions about plaque, the cause of caries, sugars, and their relationships to general health (*P* value > 0.05). Table 2 displays information regarding oral health knowledge among individuals with IDD compared to the control group.

**Table 2:** Oral health knowledge among individuals with developmental delay compared to the control group.

Questions	Developmental delay *n* (%)	Control *n* (%)	*P*-value
1. Dental plaque is formed by colonizing bacteria			
Yes	99 (58.9%)	90 (60.4%)	0.188
No	12 (7.1%)	4 (2.7%)	
Don't Know	57 (33.9%)	55 (36.9%)	
2. Dental caries mainly caused by the bacteria			
Yes	119 (70.8%)	117 (79.1%)	0.157
No	23 (13.7%)	18 (12.2%)	
Don't Know	26 (15.5%)	13 (8.8%)	
3. Having sugars can lead to dental caries			
Yes	148 (88.6%)	140 (94.0%)	0.109
No	12 (7.2%)	8 (5.4%)	
Don't Know	7 (4.2%)	1 (0.7%)	
4. Having soft drinks affect dental health			
Yes	142 (85.0%)	141 (94.6%)	***0*** **.** ***017*** *
No	12 (7.2%)	5 (3.4%)	
Don't Know	13 (7.8%)	3 (2.0%)	
5. Is there any relationship between oral health and overall health?			
Yes	127 (76.5%)	125 (83.9%)	0.096
No	17 (10.2%)	15 (10.1%)	
Don't Know	22 (13.3%)	9 (6.0%)	
6. It is normal for your gum to bleed during brushing			
Yes	80 (47.9%)	31 (20.8%)	***0*** **.** ***001*** *
No	76 (45.5%)	112 (75.2%)	
Don't Know	11 (6.6%)	6 (4.0%)	
7. It is normal for your gum to be red			
Yes	78 (46.4%)	49 (32.9%)	***0*** **.** ***026*** *
No	75 (44.6%)	89 (59.7%)	
Don't Know	15 (8.9%)	11 (7.4%).	
8. It is normal for your gum to be swelling			
Yes	38 (22.6%)	5 (3.4%)	***0*** **.** ***001*** *
No	119 (70.8%)	139 (93.3%)	
Don't Know	11 (6.5%)	5 (3.4%)	
9. Brushing teeth regularly protects your teeth			
Yes	145 (86.3%)	142 (95.3%)	***0*** **.** ***012*** *
No	13 (7.7%)	6 (4.0%)	
Don't Know	10 (6.0%)	1 (0.7%)	
10. You need to visit a dentist only when you have a toothache			
Yes	86 (51.2%)	32 (21.5%)	***0*** **.** ***001*** *
No	71 (42.23%)	116 (77.9%)	
Don't Know	11 (6.5%)	1 (0.7%)	
11. You need a hard toothbrush to clean your teeth			
Yes	62 (36.9%)	7 (4.7%)	***0*** **.** ***001*** *
No	95 (56.5%)	136 (91.3%)	
Don't Know	11 (6.5%)	6 (4.0%)	
12. Dental floss is necessary to keep your teeth clean			
Yes	87 (52.1%)	113 (75.8%)	***0*** **.** ***001*** *
No	57 (34.1%)	20 (13.4%)	
Don't Know	23 (13.8%)	16 (10.7%)	

* Significant result, *P* < .05.

The results of the participant's dental behavior indicated that fewer individuals in the IDD group brushed their teeth once or twice daily (83.2%), compared to the control group (93.3%) (*P* value < 0.05). Only 22.6% of the participants with IDD were capable of brushing their teeth without assistance, in contrast to the control group's 100%. In addition, the control group uses more fluoridated toothpaste than those with IDD (*P* value < 0.001). In contrast, the IDD group consumed significantly less sugary foods (*P* value < 0.05) and soda (*P* value < 0.001) than the control group. However, there was no significant difference between groups regarding the use of dental floss or the frequency of using mouthwash (*P* value > 0.05). Table 3 displays the participants’ responses to questions regarding dental behavior.

**Table 3:** Dental behavior among individuals with developmental delay compared to the control group.

Variable	Developmental delay	Control	*P-*Value
How often do you brush?			***0*** **.** ***018*** *
Once or more	139 (83.2%)	139 (93.3%)	
Occasionally	28 (16.8%)	10 (6.6%)	
Ability to brush			***0*** **.** ***001*** *
Completely without help	38 (22.6%)	149 (100%)	
Completely with help	130 (77.3%)	0 (0%)	
How often do you floss your teeth?			0.530
Once or more a day	23 (13.7%)	25 (16.8%)	
Occasionally	144 (85.7%)	124 (83.2%)	
How often do use mouth-rinse?			0.456
Once or more a day	39 (23.2%)	40 (26.8%)	
Occasionally	129 (76.8%)	109 (73.2%)	
Do you use fluoridated toothpaste?			***0*** **.** ***001*** *
Yes	83 (49.4%)	113 (75.8%)	
No	33 (19.6%)	6 (4.0%)	
Don’t know	52 (31.0%)	30 (20.1%)	
Frequency of eating sweets			***0*** **.** ***011*** *
Once or more a day	139 (82.7%)	141 (94.6%)	
Occasionally	17 (17.3%)	8 (5.4%)	
Frequency of drinking soda/day			***0*** **.** ***001*** *
1–2 cans	106 (63.1%)	147 (98.7%)	
3–4 cans	60 (35.7%)	2 (1.3%)	
None	2 (1.2%)	0 (0%)	

* Significant result, *P* < .05.

## 4 Discussion

Global Developmental delays (GDD) were reported with a prevalence between 1%–3% in children under the age of five, thus considered one of the most common conditions encountered in the pediatric clinics, with most common underlying causes linked to structural or genetic brain abnormalities (33). Recent study in Jordan, reported that the genetic causes were predominant with only 28% of the patients diagnosed with global developmental delay/intellectual disability were associated with metabolic disorders (34). However, investigations yielded broad estimates for IDD, partly attributed to variations in patient populations and diagnostic methods (35). Nevertheless, recent advancement in technology enabled more accurate diagnosis. Although, some studies reported that the causes of one-third of the cases of developmental or cognitive delay can be identified by history and examination only (4), it is of paramount importance that proper diagnosis of the GDD be established at early stage to define appropriate intervention strategies. This may prevent further invasive tests and ensures better overall outcome for both the patients and the carers (36). To our knowledge, no previous data have been tracked about the prevalence of GDD amongst Jordanian population, neither about oral health knowledge and practice among affected patients, which makes the current study a pioneering investigation about this subgroup of the population. Furthermore, Jordan does not have national database regarding developmental delays or disabilities, which makes this study particularly important.

The findings of the current study unequivocally demonstrated significant differences between the subjects with IDD and the healthy individuals regarding oral health knowledge. The delayed growth group showed lower level of knowledge in the majority of the investigated domains. Despite the fact that IDD group demonstrated comparable knowledge about the correlation between bacteria, and caries, and that both groups were aware of the significance of oral health to general health, developmental delay group had obvious deficits in knowledge concerning the association between consumption of soft drinks and oral health. Additionally, the delayed growth group could not relate the bleeding, swelling and color of gingiva to the existence of a disease. Besides, responses regarding tooth brushing frequency, consistency of tooth brush and the importance of dental flossing for good oral health were more appropriately answered by the healthy control group.

Moreover, majority of the delayed growth group also believed that visiting dentist is necessary only at the incidence of a toothache. This may lead to progression of existing oral diseases to advanced state and consequently may reduce the effectiveness of any preventive measures taken to retard the initiation of the diseases.

Apparently, the inadequate oral health knowledge amongst individuals with delayed growth disorders were partly reflected in their dental behavior, as frequency of tooth brushing was more commonly practiced by healthy individuals. However, although minority of growth delay group individuals brushed their teeth unassisted, the majority (83.3%) brushed their teeth once or twice daily. This may be attributed to lack of individuals with delayed growth the necessary manual dexterity or lack of appropriate training to brush their teeth independently (37), though showed high degree of commitment and support provided by their caregivers. This is contrary to previous study where 90% of intellectually delayed patients brushed their teeth by themselves (37). Moreover, it was noteworthy to find out that the growth delay individuals consumed sweets less frequently compared to their healthy peers. Their dietary habits could have been most likely modulated by their caregivers in an attempt to reduce the incidence of dental caries and the resultant need for treatment that imposes extra health care burden. Correlation between sugar and dental decay is well documented and disseminated oral health information (38). Surprisingly, despite the growth delay individual's knowledge about correlation between consumption of soda and dental health was not fully established, their consumption was less frequent compared to healthy individuals. This behavior may have also been controlled by caregivers and indicates satisfactory level of awareness regarding drawbacks of fizzy drinks. However, knowledge and practice do not always interrelate (38) and knowledge alone does not appear to adequately motivate desired oral health behavior. Similar contradiction between knowledge and practice was observed regarding tooth flossing. Although, oral health knowledge of growth delay individuals was inadequate, but practicing of tooth flossing was comparable to their healthy counterparts (39).

Previous literature clearly reported that children, adolescents and adults with IDD experienced poorer oral health, including more tooth loss, more severe periodontal diseases, and higher caries incidence with a lower rate of restorative care, as compared to the general population (9, 18, 24). This poorer oral health provides strong indicator on the extent of oral health inequality of IDD group compared to general population (24). Provided that oral diseases are largely preventable; this could also reflect the extent of the current health care (40). Therefore, oral health inequality gap can be significantly reduced by improving the care and providing more support and education (40).

The current study suggests that delayed growth individuals and their caregivers had inappropriate oral health knowledge and this may partially explain their poor oral health behaviors compared to healthy individuals. Lack of appropriate knowledge can lead to increased risk for oral diseases (41, 42). Inadequate oral health knowledge was considered the main risk factor for dental caries development among Saudi children aged-10–14 years (41). Besides, oral health behaviors were also correlated with oral health knowledge, therefore, it was concluded that promoting oral health knowledge is crucial for improving oral health behaviors (42). Furthermore, poor quality of life was directly associated with oral diseases in IDD group (18, 19), and oral health knowledge was found to indirectly affect the oral health related quality of life (43).

The literature lacks adequate information about the oral health knowledge and oral behaviors of the IDD individuals. The current study agrees with previous study in which adolescents with mild or border line intellectual disabilities found to visit the dentist less regularly and brush less frequently (44). The IDD group also reported using unfluoridated tooth paste less than healthy controls (38). Likewise, they showed higher rate of untreated decay and more microcavitated lesions than their peers from the general population (44). These lesions, in the absence of health-promoting behaviors such as less frequent brushing, use of unflouridated tooth paste and inability to brush teeth appropriately unassisted, are more likely to progress to advanced lesions, especially in the absence of good oral-health knowledge as demonstrated in the current study. It is believed that oral-health knowledge supplemented by oral health behaviors may prevent the progression of oral diseases and prevent the onset of these oral diseases, thus improving the quality of life (41, 42).

Gingival and periodontal diseases were the most commonly reported in IDD people (45). Needless to say, there are abundance of literature to associate periodontal inflammation with serious systemic illnesses which represent a considerable oral as well as systemic health burden (46). This subgroup of population represents a high-risk category due to frequently reported poor plaque control that predisposes to oral diseases (47). Therefore, instilling oral health knowledge and enforcing appropriate oral hygiene practices are of utmost importance. The awareness and training concerning the consequences of oral diseases have to be enhanced for caregivers, professionals and oral health services providers for adults suffering from intellectual and growth delay (48). Adopting holistic patient-centered approach in undergraduate and postgraduate curriculum was also recommended (49).

Although the current study employed reliable questionnaire, it could have been further enriched if data were obtained from direct interviews with the caregivers. Also, recruited participants were institutionalized and not residing at home under their caregiver's direct care, thus comparison between the institutionalized and home residing individuals with IDD was not possible. Furthermore, the views presented are mostly those of the caregivers not of the individuals with delayed growth. Further research is warranted to explore the views of the affected individuals themselves and the correlation between level of knowledge and the status of oral health. Another limitation can be the unmatched gender distribution of the participants recruited in the study. Hence comparison based on gender could be of minimum strength. Previous studies demonstrated gender based differences (50, 51). Notwithstanding these limitations, to our knowledge this is the first research that reports on the oral health knowledge and oral health behavior among people with IDD in Jordan.

## Data Availability

The original contributions presented in the study are included in the article/supplementary material, further inquiries can be directed to the corresponding authors.

## References

[1] Verma SK Jha AK Prakash O Ekram S Tigga C Noorani MK Impact of dental and orofacial trauma on oral health-related quality of life in adults: a systematic review. Chin J Traumatol. (2024) 27(5):249–53. 10.1016/j.cjtee.2023.05.003 37344288 PMC11401488

[2] Rosa A Pujia AM Arcuri C. The protective role antioxidant of vitamin C in the prevention of oral disease: a scoping review of current literature. Eur J Dent. (2024) 18(4):965–70. 10.1055/s-0044-1786845 39013452 PMC11479726

[3] Rosa A Pujia AM Arcuri C. Investigation of alveolar osteitis and the effectiveness of laser treatment: a unified meta-analysis and review of the literature. BMC Oral Health. (2024) 24:700. 10.1186/s12903-024-04461-w 38886713 PMC11184811

[4] Mithyantha R Kneen R McCann E Gladstone M. Current evidence-based recommendations on investigating children with global developmental delay. Arch Dis Child. (2017) 102(11):1071–6. 10.1136/archdischild-2016-311271 29054862 PMC5738593

[5] Choo YY Agarwal P How CH Yeleswarapu SP. Developmental delay: identification and management at primary care level. Singapore Med J. (2019) 60(3):119. 10.11622/smedj.2019025 30997518 PMC6441684

[6] Allerton LA Welch V Emerson E. Health inequalities experienced by children and young people with intellectual disabilities: a review of literature from the United Kingdom. J Intellect Disabil. (2011) 15(4):269–78. 10.1177/1744629511430772 22129526

[7] Emerson E Baines S. Health inequalities and people with learning disabilities in the UK. Tizard Learn Disabil. (2011) 16(1):42–48. 10.5042/tldr.2011.0008

[8] Merrick J Merrick E. Equal treatment: closing the gap. A formal investigation into physical health inequalities experienced by people with learning disabilities and/or mental health problems. J Policy Pract Intellect Disabil. (2007) 4(1):73. 10.1111/j.1741-1130.2006.00100.x

[9] Anders PL Davis EL. Oral health of patients with intellectual disabilities: a systematic review. Spec Care Dentist. (2010) 30(3):110–7. 10.1111/j.1754-4505.2010.00136.x 20500706

[10] Wilson NJ Lin Z Villarosa A George A. Oral health status and reported oral health problems in people with intellectual disability: a literature review. J Intellect Develop Disabil. (2019) 44(3):292–304. 10.3109/13668250.2017.1409596

[11] Aida J Kondo K Yamamoto T Hirai H Nakade M Osaka K Oral health and cancer, cardiovascular, and respiratory mortality of Japanese. J Dent Res. (2011) 90(9):1129–35. 10.1177/0022034511414423 21730255

[12] Cohen W Rose LF Minsk L. The periodontal–medical risk relationship. Compend Contin Educ Dent. (2001) 22(2 Spec No):7–11. 19248251

[13] Genco RJ Glurich I Haraszthy V Zambon J DeNardin E. Overview of risk factors for periodontal disease and implications for diabetes and cardiovascular disease. Compend Contin Educ Dent (Jamesburg, NJ: 1995). (2001) 22(2 Spec No):21–3. Wilson et al. BMC Public Health (2019) 19:1530 Page 14 of 16. 19248254

[14] Joshipura KJ Hung H-C Rimm EB Willett WC Ascherio A. Periodontal disease, tooth loss, and incidence of ischemic stroke. Stroke. (2003) 34(1):47–52. 10.1161/01.STR.0000052974.79428.0C 12511749

[15] Jung SH Ryu JI Jung DB. Association of total tooth loss with socio-behavioural health indicators in Korean elderly. J Oral Rehabil. (2011) 38(7):517–24. 10.1111/j.1365-2842.2010.02178.x 21118289

[16] Tada A Miura H. Prevention of aspiration pneumonia (AP) with oral care. Arch Gerontol Geriatr. (2012) 55(1):16–21. 10.1016/j.archger.2011.06.029 21764148

[17] Walls AW Steele JG. Geriatric oral health issues in the United Kingdom. Int Dent J. (2001) 51(3 Suppl):183–7. 10.1002/j.1875-595X.2001.tb00865.x 11561877

[18] Alves NS Gavina VP Cortellazzi KL Antunes LA Silveira FM Assaf AV. Analysis of clinical, demographic, socioeconomic, and psychosocial determinants of quality of life of persons with intellectual disability: a crosssectional study. Spec Care Dentist. (2016) 36(6):307–14. 10.1111/scd.12196 27545115

[19] Couto P Pereira PA Nunes M Mendes RA. Oral health-related quality of life of Portuguese adults with mild intellectual disabilities. PLoS One. (2018) 13(3):e0193953. 10.1371/journal.pone.0193953 29561892 PMC5862473

[20] Hillebrecht AL Hrasky V Anten C Wiegand A. Changes in the oral healthrelated quality of life in adult patients with intellectual disabilities after dental treatment under general anesthesia. Clin Oral Investig. (2019) 23(10):3895–903. 10.1007/s00784-019-02820-4 30707300

[21] Locker D. “Measuring oral health and quality of life”. In: Slade GD, editor. Concepts of Oral Health, Disease and the Quality of Life. Chapel Hill, NC: Deaprtment of Dental Ecology, School of Dentistry, University of North Carolina (1997). p. 11–24. Available online at: https://www.adelaide.edu.au/arcpoh/downloads/publications/reports/miscellaneous/measuring-oral-health-and-quality-of-life.pdf.

[22] Zucoloto ML Maroco J Campos JA. Impact of oral health on health-related quality of life: a cross-sectional study. BMC Oral Health. (2016) 16(1):55. 10.1186/s12903-016-0211-2 27176473 PMC4866408

[23] McGrath C Zhou N Wong HM. A systematic review and meta-analysis of dental plaque control among children and adolescents with intellectual disabilities. J Appl Res Intellect Disabil. (2019) 32(3):522–32. 10.1111/jar.12561 30734986

[24] Zhou N Wong HM Wen YF McGrath C. Oral health status of children and adolescents with intellectual disabilities: a systematic review and metaanalysis. Dev Med Child Neurol. (2017) 59(10):1019–26. 10.1111/dmcn.13486 28627071

[25] Okoro CA Hollis ND Cyrus AC Griffin-Blake S. Prevalence of disabilities and health care access by disability status and type among adults—united States, 2016. MMWR Morb Mortal Wkly Rep. (2018) 67(32):882–7. 10.15585/mmwr.mm6732a3 30114005 PMC6095650

[26] AAID. Definition of Iintellectual Ddisability. Silver Spring, (MD): American Association on Intellectual Disabilities (2019. Available online at: http://aaidd.org/intellectual-disability/definition#.Wwg2OKkh0dU.

[27] Zablotsky B Black LI Maenner MJ Schieve LA Danielson ML Bitsko RH Prevalence and trends of developmental disabilities among children in the United States: 2009-2017. Pediatrics. (2019) 144(4):e20190811. 10.1542/peds.2019-0811 31558576 PMC7076808

[28] Waldron C Nunn J Mac Giolla Phadraig C Comiskey C Guerin S van Harten MT Oral hygiene interventions for people with intellectual disabilities. Cochrane Database Syst Rev. (2019) 5(5):CD012628. 10.1002/14651858.CD012628.pub2 31149734 PMC6543590

[29] Bélanger SA Caron J. Evaluation of the child with global developmental delay and intellectual disability. Paediatr Child Health. (2018) 23(6):403–19. 10.1093/pch/pxy093 30919832 PMC6234423

[30] Meurs D Rutten M de Jongh A. Does information about patients who are intellectually disabled translate into better cooperation during dental visits? Spec Care Dentist. (2010) 30(5):200–5. 10.1111/j.1754-4505.2010.00152.x 20831738

[31] Thole K Chalmers J Ettinger RL Warren J. Iowa Intermediate care facilities: an evaluation of care providers’attitudes toward oral hygiene care. Spec Care Dentist. (2010) 30(3):99–105. 10.1111/j.1754-4505.2010.00131.x 20500704

[32] The World Medical Association. WMA declaration of Helsinki—ethical Pprinciples for 422 Mmedical Rresearch Iinvolving Hhuman Ssubjects Aadopted by the 18th WMA Ggeneral 423 Aassembly, Helsinki, Finland (1964). Available online at: https://www.wma.net/policies-post/wma-declaration-of-helsinki-ethical-principles-for-medical-research-involving-human-subjects.

[33] Majnemer A Shevell MI. Diagnostic yield of the neurologic assessment of the developmentally delayed child. J Pediatr. (1995) 127:193–9. 10.1016/S0022-3476(95)70294-6 7543566

[34] Masri AT Oweis L Ali M Hamamy H. Global developmental delay and intellectual disability in the era of genomics: diagnosis and challenges in resource limited areas. Clin Neurol Neurosurg. (2023) 230:107799. 10.1016/j.clineuro.2023.107799 37236004

[35] Shevell M Ashwal S Donley D Flint J Gingold M Hirtz D Practice parameter: evaluation of the child with global developmental delay: report of the quality standards subcommittee of the American academy of neurology and the practice committee of the child neurology society. Neurology. (2003) 60:367–80. 10.1212/01.WNL.0000031431.81555.16 12578916

[36] Moeschler JB Shevell M. Comprehensive evaluation of the child with intellectual disability or global developmental delays. Pediatrics. (2014) 134:e903–18. 10.1542/peds.2014-1839 25157020 PMC9923626

[37] Bissar AR Kaschke I Schulte AG. Oral health in 12- to 17-year-old athletes participating in the German special olympics. Int J Paediatr Dent. (2010) 20(6):451–7. 10.1111/j.1365-263X.2010.01065.x 20642469

[38] Van Loveren C. Sugar restriction for caries prevention: amount and frequency. Which is more important? Caries Res. (2019) 53(2):168–75. 10.1159/000489571 30089285 PMC6425816

[39] Lakshmi KPD Venkatalakshmi S Bharath C Saravanan N Reddy LS Nagilla J. Correlation of knowledge, attitude, and practice with their oral health Status among young adults of nursing care: a cross-sectional survey. J Pharm Bioallied Sci. (2022) 14(Suppl 1):S82–6. 10.4103/jpbs.jpbs_555_21 36110659 PMC9469441

[40] Ward LM Cooper SA Hughes-McCormack L Macpherson L Kinnear D. Oral health of adults with intellectual disabilities: a systematic review. J Intellect Disabil Res. (2019) 63(11):1359–78. 10.1111/jir.12632 31119825

[41] Amin TT Al-Abad BM. Oral hygiene practices, dental knowledge, dietary habits and their relation to caries among male primary school children in al hassa, Saudi Arabia. Int J Dent Hyg. (2008) 6:361–70. 10.1111/j.1601-5037.2008.00310.x) 19138188

[42] Zheng S Zhao L Ju N Hua T Zhang S Liao S. Relationship between oral health-related knowledge, attitudes, practice, self-rated oral health and oral health-related quality of life among Chinese college students: a structural equation modeling approach. BMC Oral Health. (2021) 21:1–11. 10.1186/s12903-021-01419-0) 33676475 PMC7936478

[43] Zhao J Shi H Wang J Huang R Liu Y Zhang Y Association of oral health knowledge, self-efficacy and behaviours with oral health-related quality of life in Chinese primary school children: a cross-sectional study. BMJ Open. (2022) 12(12):e062170. 10.1136/bmjopen-2022-062170 36521895 PMC9756180

[44] Vermaire JH Kalf SM Schuller AA. Oral health and oral health behaviour of adolescents with mild or borderline intellectual disabilities compared with a national representative sample of 17-year-olds in The Netherlands. J Appl Res Intellect Disabil. (2021) 34(2):615–23. 10.1111/jar.12829 33169895 PMC7894337

[45] Anusha D Kengadaran S Prabhakar J MuthuKrishnan K Katuri LS Vigneshwari SK Prevalence of dental caries and gingivitis among children with intellectual disability in India. J Family Med Prim Care. (2022) 11(6):2351–5. 10.4103/jfmpc.jfmpc_655_21 36119303 PMC9480631

[46] Hsieh K Murthy S Heller T Rimmer JH Yen G. Reported gum disease as a cardiovascular risk factor in adults with intellectual disabilities. J Intellect Disabil Res. (2018) 62(3):187–98. 10.1111/jir.12438 29114946

[47] Morgan JP Minihan PM Stark PC Finkelman MD Yantsides KE Park A The oral health status of 4,732 adults with intellectual and developmental disabilities. J Am Dent Assoc. (2012) 143:838–46. 10.14219/jada.archive.2012.0288 22855898 PMC4527687

[48] Cumella S Ransford N Lyons J Burnham H. Needs for oral care among people with intellectual disability not in contact with community dental services. J Intellect Disabil Res. (2000) 44(Pt 1):45–52. 10.1046/j.1365-2788.2000.00252.x 10711649

[49] Blaizot A Catteau C Delfosse C Hamel O Trentesaux T. Obstacles to comprehensive dental care in patients with sustained limitations of their decision-making abilities: findings from a delphi study. Eur. J. Oral Sci. (2018) 126:222–33. 10.1111/eos.12413 29676806

[50] Lindemann R Zaschel-Grob D Opp S Lewis MA Lewis C. Oral health status of adults from a California regional center for developmental disabilities. Spec Care Dentist. (2001) 21(1):9–14. 10.1111/j.1754-4505.2001.tb00217.x 11795453

[51] Abu-Gharbieh E Saddik B El-Faramawi M Hamidi S Basheti M Basheti M. Oral health knowledge and behavior among adults in the United Arab Emirates. Biomed Res Int. (2019) 2019:7568679. 10.1155/2019/7568679 30881996 PMC6381549

